# *Pseudomonas aeruginosa* responds to exogenous polyunsaturated fatty acids (PUFAs) by modifying phospholipid composition, membrane permeability, and phenotypes associated with virulence

**DOI:** 10.1186/s12866-018-1259-8

**Published:** 2018-09-14

**Authors:** Lyssa Y. Baker, Chelsea R. Hobby, Andrew W. Siv, William C. Bible, Michael S. Glennon, Derek M. Anderson, Steven J. Symes, David K. Giles

**Affiliations:** 1 Department of Biology, Geology, and Environmental Science, The University of Tennessee at Chattanooga, Chattanooga, TN USA; 2 Department of Chemistry and Physics, The University of Tennessee at Chattanooga, Chattanooga, TN USA

**Keywords:** *Pseudomonas aeruginosa*, Fatty acids, Phospholipids, Antimicrobial peptides, Biofilm, Motility

## Abstract

**Background:**

*Pseudomonas aeruginosa*, a common opportunistic pathogen, is known to cause infections in a variety of compromised human tissues. An emerging mechanism for microbial survival is the incorporation of exogenous fatty acids to alter the cell’s membrane phospholipid profile. With these findings, we show that exogenous fatty acid exposure leads to changes in bacterial membrane phospholipid structure, membrane permeability, virulence phenotypes and consequent stress responses that may influence survival and persistence of *Pseudomonas aeruginosa*.

**Results:**

Thin-layer chromatography and ultra performance liquid chromatography / ESI-mass spectrometry indicated alteration of bacterial phospholipid profiles following growth in the presence of polyunsaturated fatty acids (PUFAs) (ranging in carbon length and unsaturation). The exogenously supplied fatty acids were incorporated into the major bacterial phospholipids phosphatidylethanolamine and phosphatidylglycerol. The incorporation of fatty acids increased membrane permeability as judged by both accumulation and exclusion of ethidium bromide. Individual fatty acids were identified as modifying resistance to the cyclic peptide antibiotics polymyxin B and colistin, but not the beta-lactam imipenem. Biofilm formation was increased by several PUFAs and significant fluctuations in swimming motility were observed.

**Conclusions:**

Our results emphasize the relevance and complexity of exogenous fatty acids in the membrane physiology and pathobiology of a medically important pathogen. *P. aeruginosa* exhibits versatility with regard to utilization of and response to exogenous fatty acids, perhaps revealing potential strategies for prevention and control of infection.

**Electronic supplementary material:**

The online version of this article (10.1186/s12866-018-1259-8) contains supplementary material, which is available to authorized users.

## Background

The prevalent opportunistic pathogen *Pseudomonas aeruginosa* displays an array of virulence factors and antibiotic resistance mechanisms that make it a significant public health concern, particularly for cystic fibrosis patients, burn victims and nosocomial infections. The ability of *P. aeruginosa* to thrive in diverse environments can be partially explained by its tremendous metabolic capabilities, allowing utilization of numerous substrates [1, 2]. Many of these molecules have been identified to be nutritional cues, serving as signals to guide bacterial behavior (motility, biofilm, antibiotic resistance) [3–5].

An emerging concept is that some bacteria have evolved to recognize lipid molecules in a manner that affects physiology and pathogenesis [6–8]. *Vibrio* species, for example, have been shown to incorporate exogenous fatty acids into membrane phospholipids [9, 10], causing alterations in phospholipid structure and changes in biofilm formation and motility [11]. Similar findings have been found with *Acinetobacter baumannii* and *Klebsiella pneumoniae* [12, 13]. The consequences of these modifications to membrane lipids are unknown. The effects of exogenous fatty acids on bacterial growth and virulence have been demonstrated in both Gram-negative and Gram-positive bacteria. Fatty acids have influenced motility, gene expression and survival [14–18], while inhibitory action of polyunsaturated fatty acids have been documented in several pathogens [19–21].

The mechanism for fatty acid uptake and utilization, originally studied in *E. coli*, operates by a protein dependent pathway. Exogenous fatty acids are recognized by an outer membrane protein FadL which allows transport into the periplasm [22]. FadD, an inner membrane protein, intercepts the protonated long chain fatty acids and converts them into acyl-CoA thioesters (LCFA-acyl-CoA). These products of FadD have three known fates: they can be used as transcriptional regulators, metabolized by beta oxidation, or converted to phospholipids to be incorporated into the membrane [23, 24].

Several studies have begun to establish a link between fatty acid degradation and virulence in *P. aeruginosa*. Initial studies with *P. putida* and *P. fragi* established the wide range of fatty acid substrates recognized by a dual FadD system [25]. In cystic fibrosis patients, the expression of *fadD*1 and *fadD*2 was demonstrated during lung infection, suggesting a valuable role of lipid acquisition in vivo [26]. The same study strengthened the connection between Fad and virulence by further characterizing the *fadD* genes and demonstrating that inactivation of either or both transcripts led to deficiencies in fatty acid and phosphatidylcholine utilization, variation in both swimming and swarming motility and a decrease in in vivo fitness using a mouse lung infection model [26]. Moreover, the discovery of a long-chain fatty acid sensor, PsrA, by Kang et al. [14, 27] lends support to the idea of fatty acids as important signaling molecules that affect environmental and host survival.

Many studies have lauded the biological benefits of long-chain polyunsaturated fatty acids (PUFAs) to human development and health [28, 29]. The effects include structural roles within membranes, bioconversion into signaling molecules, and contributions in membrane-associated processes [30–32]. The contributions of PUFAs to membrane structure, protein function and gene regulation support further research in organisms displaying an expanded ability for fatty acid metabolism. In a *P. aeruginosa* lung infection mouse model, dietary supplementation with (n-3) PUFAs led to lower mortality, as well as relief from fluid accumulation and excessive mucin secretion [33, 34]. The same PUFAs were later shown to positively affect the outcome of pulmonary infection in mice by modulating pro- and anti-inflammatory cytokines [35].

The current study investigates the changes in lipid profile in *P. aeruginosa* upon exposure to polyunsaturated fatty acids (PUFAs) and seeks to identify if such membrane modifications cause virulence-associated responses. Growth in the presence of individual PUFAs dramatically altered phospholipid profiles and variably impacted membrane permeability, biofilm formation, motility and susceptibility to antimicrobial peptides. Overall, our data underscore the importance of fatty acids in the lifestyle of pathogenic bacteria.

## Methods

### Bacterial strains and growth conditions

The laboratory strain *Pseudomonas aeruginosa* PAO1 used in this study was obtained from the Colin Manoil, University of Washington, Seattle (originating from B. Iglewski, University of Rochester Medical Center) [36]. G56 minimal medium (0.4% Glucose, 0.4% casamino acids, 150 mM NaCl served as the growth medium unless described otherwise. A concentration of 300 μM was used for each fatty acid (Cayman Chemicals).

### Bacterial lipid extraction and thin-layer chromatography

The method of Bligh [37] and Dyer was used to extract phospholipids from 14 ml of bacterial culture. Isolated phospholipids were deposited onto Silica Gel 60 TLC plates and subsequently separated using a solvent system consisting of chloroform, methanol and acetic acid (65:25:10 *v*/v/v). Visualization of phospholipids was achieved by charring plates with a solution of 10% sulfuric acid in 100% ethanol followed by heating to 150 °C for approximately 1 m. Plates were scanned using a Canon CanoScan 9000F.

### Ultra performance liquid chromatography/ESI-mass spectrometry

The methodology for analysis of extracted bacterial phospholipids can be found in previous publications [12, 13]. Briefly, the extracted phospholipids were prepared as a 300 ppm (total lipid) sample. Diluent consisted of a 50:50 mixture of solvents A and B where A = 30:70 25 mM pH 6.7 ammonium acetate:methanol and B = methanol. Chromatographic separation invloved gradient elution on an ACQUITY UPLC system (Waters, Milford, MA) equipped with a BEH C8 column (2.1 × 100 mm; 1.7 um particles). Detection of analytes was achieved by quadrupole mass spectrometry following electrospray ionization in the negative mode.

### Crystal violet uptake assay

The crystal violet assay was performed as previously described [11–13]. Briefly, 7 ml cultures of *P. aeruginosa* in G56 minimal media were grown in the presence and absence of 300 μM each PUFA to logarithmic phase. Cultures were pelleted, washed and resuspended in 5 ml PBS at an OD of 0.5. Every 5 m following addition of crystal violret (5 μg/ml), 1 ml was removed, pelleted, and the supernatant was measured at OD_590_. Inclusion of a control (containing CV but no bacteria) allowed normalization of the data. The amount of CV detected (representing dye not taken up) was converted to percentage of uptake using Excel. Three biological replicates were performed and all standard deviations were calculated to be less than 5% (not graphed for visual clarity).

### Ethidium bromide accumulation and exclusion assays

Methodology for measurement of the ethidium bromide excluded from bacteria has been previously published [13]. Briefly, cultures of *P. aeruginosa* were grown to logarithmic phase, pelleted, and resuspended at an OD of 0.7. Every 5 m following addition of ethidium bromide (5 μg/ml), 1 ml of culture was removed, pelleted, and the supernatant was analyzed using an excitation wavelength of 530 nm and detection wavelength of 585 nm. A control sample (containing EtBr but no bacteria) allowed normalization of the data and represents the maximal fluorescence intensity (measured as 420 nm and plotted as the ‘zero’ time for each sample). The measurements were performed on a Varian Cary Eclipse Fluorescence Spectrophotometer with a 20-nm excitation slit width. Three biological replicates were performed. For measurement of accumulation of ethidium bromide, *P. aeruginosa* was grown in CM9 to logarithmic phase (OD_600_ = 0.8) in the presence and absence of 300 μM each PUFA. Bacteria were pelleted, washed with PBS and resuspended in PBS at an OD_600_ = 0.4. After addition of 20 μM of ethidium bromide, fluorescence measurements were taken every 5 m for 1 h using a Varian Cary Eclipse Fluorescence Spectrophotometer with a 10-nm excitation slit width (excitation wavelength of 545 nm, detection wavelength of 600 nm). Two biological replicates were performed.

### Biofilm assay

Biofilm formation was measured using a previously published method by O’Toole [38]. Briefly, *P. aeruginosa* cultures were prepared in 96-well microtiter plates containing G56 minimal medium supplemented with or without each fatty acid. Planktonic cells were removed after an incubation period of 20 h at 37 °C and the plates were gently washed. Crystal violet (0.1%) was added to each well and incubated for 15 m at room temperature. The plate was washed and allowed to dry. 30% acetic acid was added, allowed to incubate for 15 m, and the solubilized crystal violet was transferred to a fresh microtiter plate and measured at OD_590_. Three biological replicates were performed in octuplet and statistical significance was determined using the Students t-Test (2-tailed, paired).

### Motility assays

Soft agar assays were prepared using M9 minimal medium (0.35% agar), supplemented with 300 μM of each fatty acid. Other supplements included 2 mM MgSO4, 0.2 mM CaCl2, 0.8% glucose, 0.075 mM thiamine and 0.4% casamino acids. 2 μl of inoculum (OD_600_ = 0.1) was pipetted into the agar and the diameters were measured after 24 h of incubation at 37 °C. Two biological replicates were performed in quadruplicate.

### Antimicrobial peptide susceptibility assay

Microtiter plate assay methodology was performed as previously published [11–13]. *P. aeruginosa* was grown to logarithmic phase in G56 or CM9. Cultures were centrifuged, washed with minimal media, and resuspended at a starting OD_600_ of 0.1 and exposed to two-fold concentrations of polymyxin B, colistin, or imipenem. Plates were incubated for 20 h at 37 °C and a Biotek Synergy microplate reader was used to read absorbance at OD_600_.

## Results

### PUFA exposure results in accelerated growth and altered phospholipid profiles of *Pseudomonas aeruginosa*

**Fig. 1:**
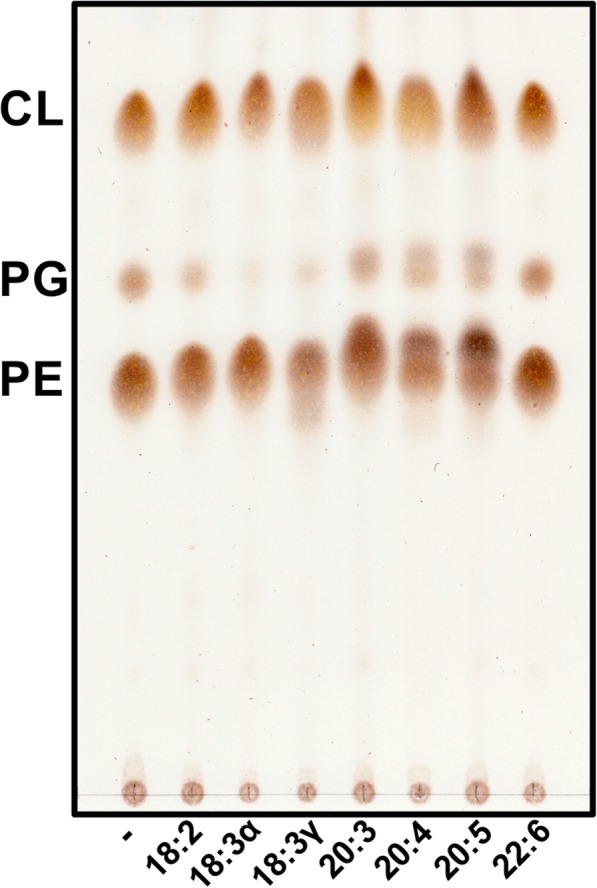
Thin-layer chromatography of phospholipids extracted from *Pseudomonas aeruginosa* grown in the presence of individual polyunsaturated fatty acids. Bacteria were grown to exponential phase (OD ≈ 0.8) in G56 minimal media at 37 °C with or without 300 μM of the indicated fatty acids (linoleic acid [18:2], alpha-linoleic acid [18:3α], gamma-linolenic acid [18:3γ], dihomo-gamma-linolenic acid [20:3], arachidonic acid [20:4], eicosapentaenoic acid [20:5] and docosahexaenoic acid [22:6]) prior to Bligh and Dyer extraction of phospholipids and separation by TLC in the solvent system chloroform/methanol/acetic acid (65:25:10 *v*/v/v). The plate was charred and scanned to produce the final image

The effect of PUFAs on *P. aeruginosa* phospholipids was investigated by growing bacteria in the presence and absence of micromolar concentrations of each fatty acid. Minimal medium was used to exclude fatty acid contributions of complex media, as are present in nutrient rich media. Growth curves indicated that each PUFA caused modest increases in growth rate during 12 h of incubation (Additional file 1: Figure S1). Furthermore, growth of *P. aeruginosa* was possible with each PUFA when provided as the sole carbon source (Additional file 2: Figure S2). The major phospholipids produced by *P. aeruginosa* [phosphatidylethanolamine (PE), phosphatidylglycerol (PG), phosphatidylcholine (PC) and cardiolipin (CL)] were analyzed by thin-layer chromatography (TLC) (Fig. 1). The phospholipid profiles reflect a structural shift indicative of incorporation of the longer chain, more unsaturated fatty acid. It is possible to distinguish two distinct species of the same phospholipid, as well as possible preference for the more hydrophobic species (see PE for 20:3, 20:4 and 20:5).

### UPLC/ESI-MS analyses indicate assimilation of exogenous PUFAs into *P aeruginosa* phospholipids

**Fig. 2:**
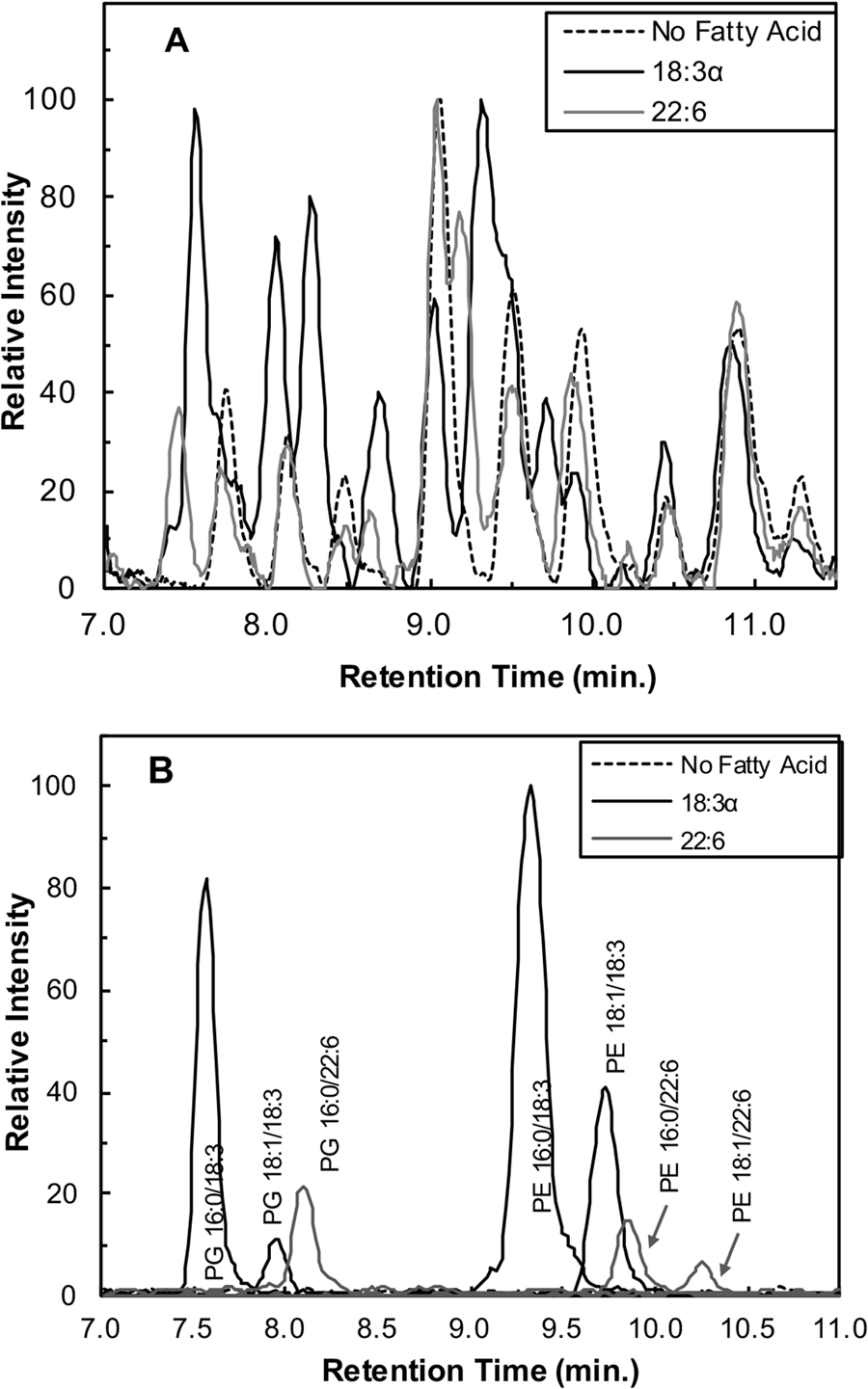
Ultra performance liquid chromatography/mass spectrometry of isolated phospholipids from *Pseudomonas aeruginosa* grown in the presence and absence of fatty acids. *P. aeruginosa* was grown with or without 300 μM of a given fatty acid at 30 °C in G56 (pH 7.4) to logarithmic phase. Lipids were extracted using the Bligh and Dyer method but included an extra wash step to increase the purity of the isolated lipids. The lipid extract was injected (5 μL) into a Waters UPLC for gradient elution using a reversed phase C-8 column. [M-H]^−^ ions were detected by quadrupole mass spectrometry following electrospray ionization. **a** Total ion chromatograms (*m/z* 650–850) comparing the control to cultures exposed to either 18:3α or 22:6 fatty acid. Changes in the chromatograms compared to the control suggest modifications of the phospholipid profile that depend on the supplied fatty acid. **b** Extracted ion chromatograms (XIC) showing new peaks, in the exposed samples, that are absent in the control. The labeled peaks are predicted using the Lipid Maps Database (http://www.lipidmaps.org/) and are based on the *m/z* of the parent ion. From left to right, the XIC’s correspond to the following *m/z* values: 743.5, 769.5, 793.5, 712.5, 738.5, 762.5, 788.5. The control was mass filtered for all of these same ions but only noise was present. As a result, only the XIC at 793.5 is used to represent the control

Ultra-performance liquid chromatography/electrospray ionization mass spectrometry (UPLC/ESI-MS) was employed to better define the qualitative changes observed by TLC. Bacterial lipids were extracted following growth with individual PUFAs and subjected to chromatographic separation using reversed-phase gradient elution. The samples produced chromatograms (Fig. 2) indicating structural changes to the phospholipid profile, yielding peaks dependent upon the specific fatty acid supplemented. Figure 2a shows representative total ion chromatograms (TIC) and extracted ion chromatograms (XIC) for *Pseudomonas* cultures grown in the presence of select fatty acids. Data for all fatty acids tested is provided in the Supplemental materials (Additional file 3: Figure S3). The introduction of new chromatographic peaks absent in the control are indicative of fatty acid-modified phospholipid species.

Similar to TLC, the LC-MS results showed abundant PE and PG species. Mass spectrometry confirmed that these phospholipids are composed of fatty acyl chains derived from the exogenously supplied fatty acid. Extracted ion chromatograms from exogenous fatty acid exposed samples revealed [M-H]^−^ ions corresponding to phospholipid species consisting of at least one acyl chain matching the supplemented fatty acid (Fig. 2b). For example, phospholipids from fatty-acid supplemented cultures had peaks corresponding to [M-H]^−^ ions of *m/z* 743.5 (16:0/18:3), 769.5 (18:1/18:3), and 793.5 (16:0/22:6), which are not present in the control (Fig. 2b; see Additional file 4: Figure S4 for mass spectra). Similarly, PE species displayed modified XIC peaks at *m/z* 712.5 (16:0/18:3), 738.5 (18:1/18:3), 762.5 (16:0/22:6), and 788.5 (18:1/22:6) (Fig. 2b). Taken together, the data supports the assimilation of exogenously supplied fatty acids into both PG and PE of *P. aeruginosa*.

### Exogenous fatty acids alter the membrane permeability of *P. aeruginosa*

**Fig. 3:**
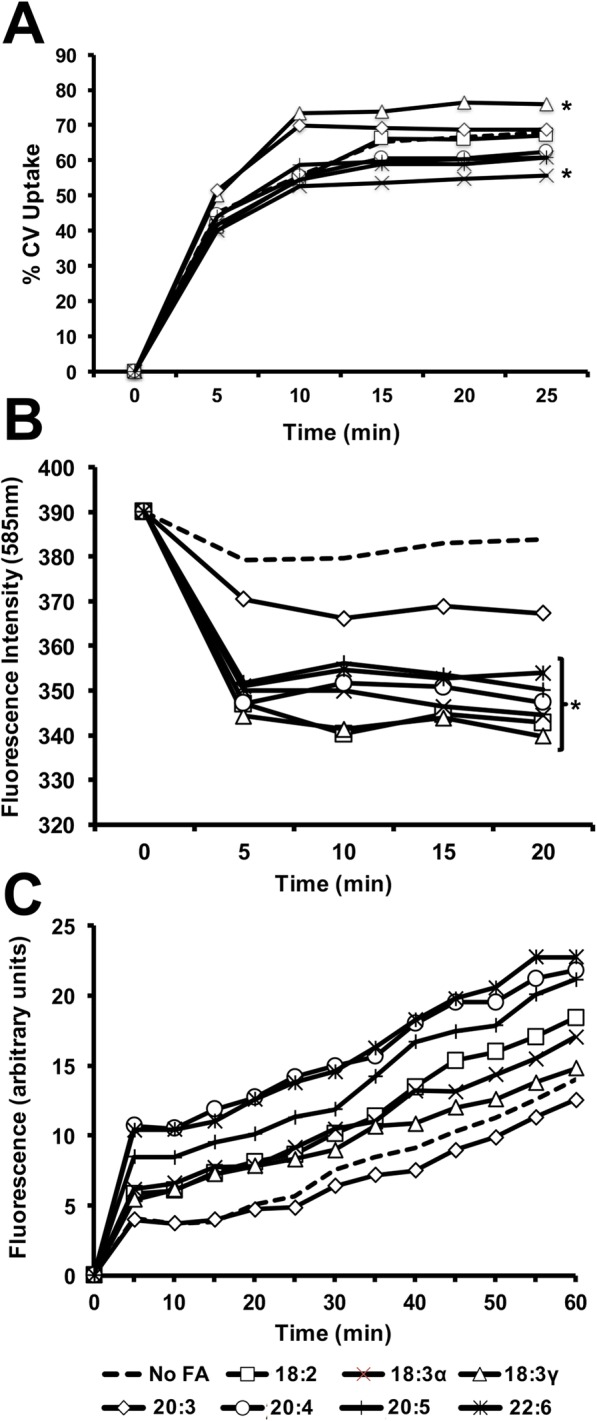
The effect of exogenous fatty acids on hydrophobic compound uptake in *Pseudomonas aeruginosa*. **a** Bacteria were grown at 37 °C in G56 (pH 7.4) with and without 300 μM of the indicated fatty acids to mid-log phase (OD = 0.8). Cultures were gently pelleted, washed with PBS and resuspended in an equal volume of PBS (OD_600_ = 0.4). The amount of CV in the supernatant following centrifugation was measured at regular intervals and expressed graphically as percentage of CV uptake. All standard deviations were less than 3% (not graphed for visual clarity). **b** Bacteria were grown at 37 °C in CM9 with and without 300 μM of the indicated fatty acids to mid-log phase (OD = 0.8). Cultures were gently pelleted, washed with PBS and resuspended in an equal volume of PBS (OD_600_ = 0.7). The amount of EtBr in the supernatant following centrifugation was measured as fluorescence emission intensity at 585 nm (excitation wavelength of 530 nm). Asterisks indicate significant difference (*, *p* < 0.001) as compared to control. **c** Bacteria were grown at 37 °C in CM9 with and without 300 μM of the indicated fatty acids to mid-log phase (OD = 0.8). Cultures were gently pelleted, washed with PBS and resuspended in an equal volume of PBS (OD_600_ = 0.4). Following addition of 20 μM EtBr, fluorescence intensity was measured every 5 m for 1 h (excitation wavelength of 550; emission wavelength of 600)

The observed alterations to membrane phospholipid structure prompted measurement of cellular membrane permeability following fatty acid exposure. As measured using a crystal violet uptake assay, the fatty acids elicited significant alterations to permeability in *P. aeruginosa* (Fig. 3). *P. aeruginosa* displayed mostly decreased membrane permeability, with the sole exception being gamma-linolenic acid. Interestingly, the omega-3 counterpart elicited the largest decrease (> 10%) in permeability. Since crystal violet is known to bind surface components (eg, sugars, proteins) of bacterial, these modest changes to permeability were further investigated by using ethidium bromide as another measure of membrane permeability. Compared to control, each fatty acid elicited lower emission intensity of ethidium bromide excluded from bacteria over the course of 20 min (Fig. 3b). Furthermore, cell-associated fluorescence was measured with or without PUFA adaptation. This accumulation assay corroborated the exclusion assay, indicating increased fluorescence for all fatty acids except dihomo-gamma-linolenic acid (20:3) (Fig. 3c). Collectively, this data suggests that incorporation of PUFAs increases *P. aeruginosa* membrane permeability.

### Exogenous fatty acids influence antimicrobial peptide MIC in *P. aeruginosa*

**Fig. 4:**
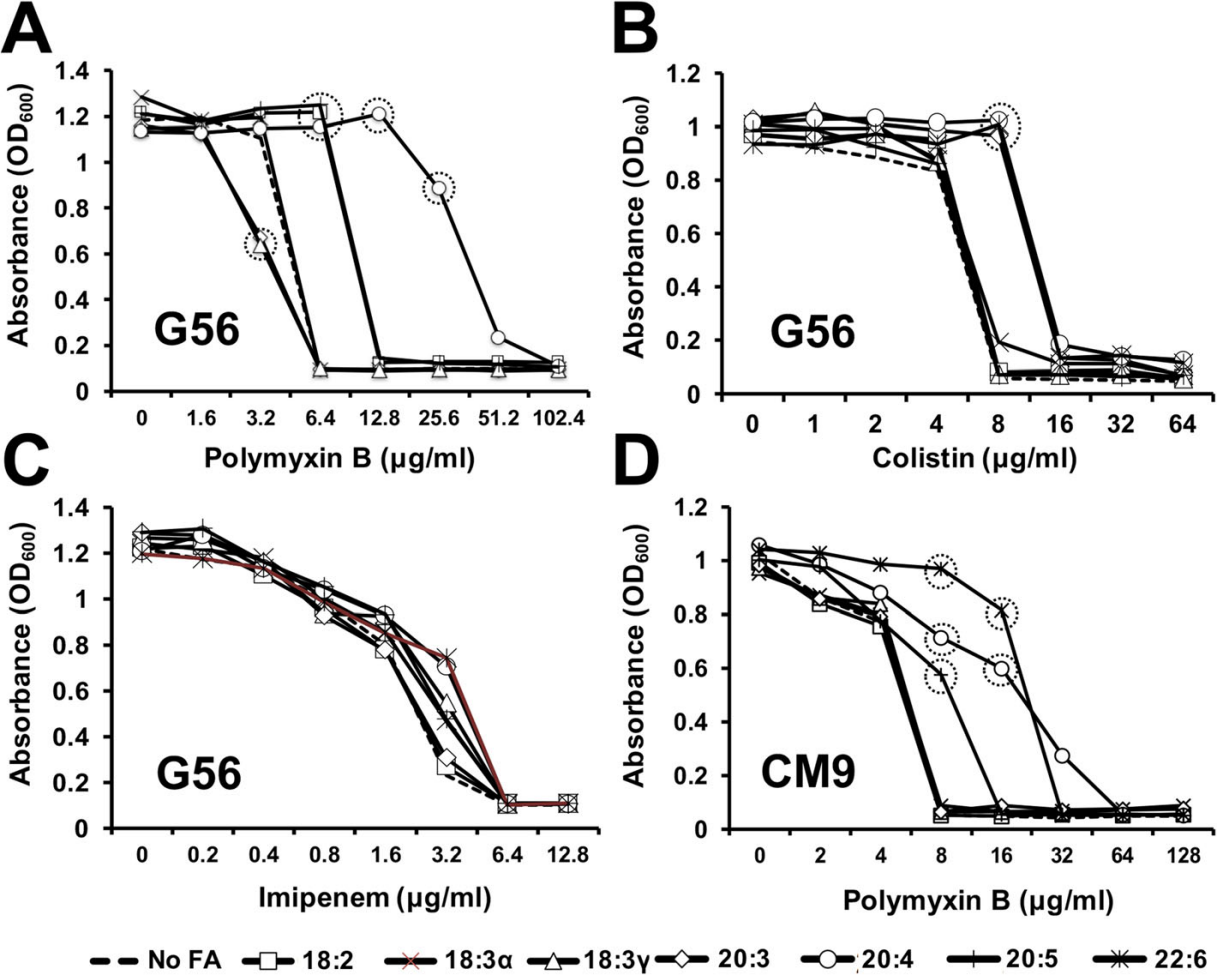
The effect of exogenous fatty acids on polymyxin B, colistin, and imipenem resistance in *Pseudomonas aeruginosa*. Bacteria were grown at 37 °C in G56 (pH 7.4) (**a**-**c**) or CM9 (**d**) with and without 300 μM of the indicated fatty acids to mid-log phase (OD = 0.8). Cultures were pelleted, washed with G56 and resuspended in G56or CM9 to an OD_600_ of 0.1. Fatty acids were added to a final concentration of 300uM. The bacterial suspension was distributed into microtiter plates and two-fold concentrations of polymyxin B, colistin, or imipenem were added. After 20 h incubation at 37 °C, the optical density (600 nm) was read using a Biotek Synergy microplate reader. Experiments were conducted in triplicate, with each value representing the mean and standard deviation. Symbols circled by dotted line indicate significant differences (*p* < 0.002) as compared to the control (no fatty acid) at the particular antimicrobial concentration

Since many antimicrobials target membrane integrity, we hypothesized that phospholipid incorporation of exogenous fatty acids would alter the bacterial response to some antimicrobials. We examined the effects of fatty acids on the MIC of two cyclic peptide antibiotics polymyxin B and colistin resistance using a microtitre plate assay (Fig. 4a and b). The assays were performed following growth in the presence of 300 μM fatty acid, with continued fatty acid availability during assay incubation. Several fatty acids (18:2, 20:4 and 20:5) led to an increase in polymyxin B MIC. Strikingly, arachidonic acid caused an 8-fold increase in MIC of polymyxin B. In another minimal medium (CM9), the increase for arachidonic acid held; however, the MIC for docosahexaenoic acid was increased 4-fold (Fig. 4d). Three fatty acids (18:3γ, 20:4 and 22:6) conferred two-fold protection against colistin. No significant difference was observed when the beta-lactam imipenem was used (Fig. 4c).

### Exogenous PUFAs affect swimming motility in *P. aeruginosa*

**Fig. 5:**
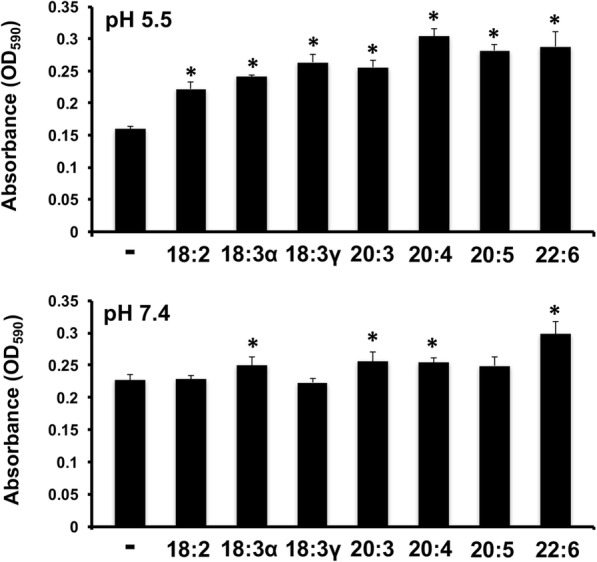
The effect of exogenous polyunsaturated fatty acids on motility of *Pseudomonas aeruginosa*. Marine broth (0.35% agar) motility plates were prepared, supplemented with 300 μM of the appropriate fatty acid or absence thereof (after cooling to 55 °C). 1 μL of inoculum (OD_600_ = 0.1) was pipetted into motility plates and observed after 24 h incubation at 37 °C. Each value represents the mean and standard deviation of two separate biological replicates performed in quadruplicate

Motility represents another phenotype associated with virulence in *P. aeruginosa* infections. Small molecules (e.g., fatty acids) are common mediators of bacterial behavior, such as chemotaxis and quorum sensing [39–41]. Furthermore, there have been many observed effects of fatty acids on bacterial motility [15, 42–44]. *P. aeruginosa* motility was measured in response to individual polyunsaturated fatty acids by inoculating bacteria into motility plates prepared with M9 minimal media (0.2% glucose, 0.4% casamino acids) and supplemented with 0.4% agar and 300 μM of each fatty acid. The soft agar assays revealed significant decreases in motility for all but one fatty acid (20:3) (Fig. 5). Arachidonic and eicosapentaenoic acids elicited a nearly 50% decrease in swimming motility.

### PUFAs influence biofilm formation in *P. aeruginosa*

**Fig. 6:**
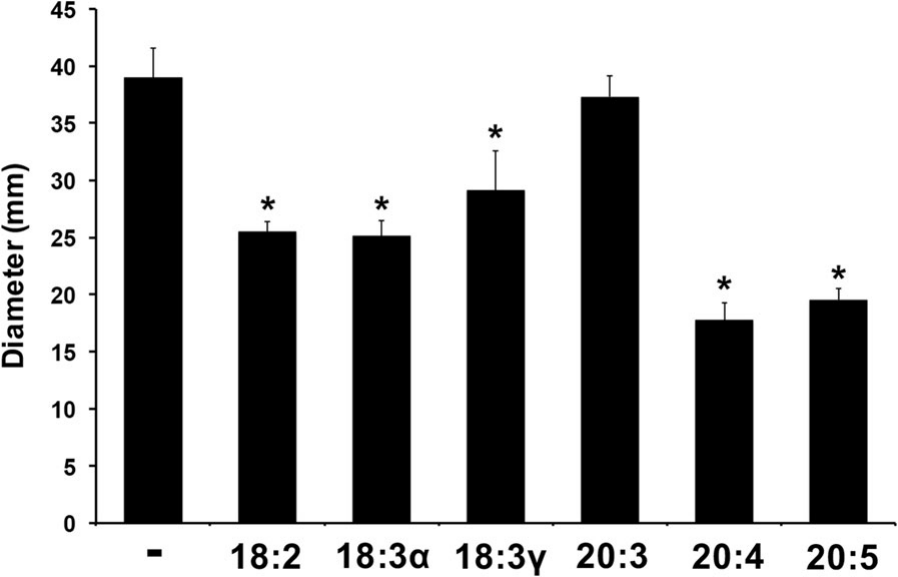
Incubation with exogenous fatty acids alters biofilm formation in *Pseudomonas aeruginosa*. Overnight cultures were pelleted, washed, resuspended in appropriate media and inoculated onto microtiter plates (starting OD ~ 0.1) in octuplet. Each culture was grown in the presence of 300 μM of the indicated fatty acids. After 24 h incubation, the biofilm assay by O’Toole was performed in media buffered at two pH levels (5.5 and 7.4). The absorbance (OD_590_) was measured using a Biotek Synergy microplate reader. Each value represents the mean and standard deviation of 8 wells. Asterisks indicate *p*-values determined to be less than 0.008 when compared to the no fatty acid controls

Formation of biofilms confers advantages to bacteria. The effects of fatty acids on biofilm formation in *P. aeruginosa* was measured using a crystal violet assay, with bacteria grown in the presence of fatty acid for 24 h. The capacity for biofilm formation was tested at physiological pH (7.4) and a lower pH (5.5) to mimic other *P. aeruginosa* infection sites (skin/burn infections). Addition of PUFAs increased biofilm formation in *P. aeruginosa* (Fig. 6). The same phenomenon was further amplified when the assay was performed at a lower pH (5.5).

## Discussion

As microbial resistance strategies emerge, it is important to identify novel ways to target vulnerabilities. It is becoming evident that some bacteria have broader capabilities with regard to membrane lipid homeostasis. In addition to altering de novo membrane phospholipid composition in response to environmental stress/stimuli (homeoviscous adaptation), incorporation of exogenous fatty acids constitutes another mechanism by which bacteria capitalize on their surroundings. The current study further defines and expands the fatty acid handling of *P. aeruginosa*, demonstrating structural modifications to phospholipids as well as behavioral changes associated with fatty acid exposure. Considering the pathogenic nature of *P. aeruginosa*, it is tempting to regard fatty acids as important niche-specific nutrients, signaling molecules and/or membrane modifiers. During infection, bacteria encounter free fatty acids [45–48] or liberate fatty acids by enzymatic cleavage of larger lipids. We chose a micromolar concentration (300 μM) to examine the effects of fatty acids on growth. Given the multiple infection sites inhabited by *P. aeruginosa*, the physiological relevance of 300 μM could be dependent upon the site of colonization. We have not verified bacterial responses to other concentrations of exogenous fatty acids.

The current study contributes to recent reports documenting the expanding lipid handling ability of *P. aeruginosa*. Not long after the fatty acid sensor PsrA was discovered [14], multiple FadD acyl CoA synthetase homologs were described in *P. aeruginosa* [26]. More recently, the identification of the PA3286 shunt [49] and ExFadLO [50] illustrate the expanded fatty acid capabilities possessed by *P. aeruginosa*. A bioinformatic survey of several *Pseudomonas* species reveals significant dedication of proteins predicted to be involved in the uptake and assimilation of exogenous fatty acids into phospholipids (Table 1). The conservation of this machinery among pseudomonads may underscore the adaptation to environments (soil, plant, human) offering a plethora of fatty acids.

**Table 1:** *Pseudomonas aeruginosa* possesses an expanded repertoire of genes involved in fatty acid uptake

	FadL	FadD	PlsB/PlsC	PlsX/PlsY
*P. aeruginosa PAO1*	*PA1288 PA1764, PA4589*	*PA3300, PA3299, PA3924, PA4198, PA2557, PA1617, PA3860,*	*PA3673 /PA3267, PA0358*	*PA2969 /PA0581*
*P. putida S12*	*RPPX_15880, RPPX_25840*	*RPPX_02635, RPPX_02630, RPPX_05335, RPPX_23090, RPPX_00575, RPPX_21065*	*RPPX_15320 /RPPX_16685,RPPX_09090*	*RPPX_17030 /RPPX_07310*
*P. fluorescens* F113	*PSF113_4386*	*PSF113_1301, PSF113_1302, PSF113_3051, PSF113_3081, PSF113_3882, PSF113_0964*	*PSF113_1116/PSF113_4376*	*PSF113_4149/PSF113_5367*
*P. syringae* B728a	*Psyr_3669*	*Psyr_3836, Psyr_3834, Psyr_0990*	*Psyr_1328 / Psyr_1586, Psyr_0009*	*Psyr_1645 / Psyr_4637*

*Pseudomonas aeruginosa* was bioinformatically assessed for homologs to FadL, FadD and acyltransferases involved in utilization of exogenous fatty acids. The identified homologues illustrate the enhanced ability for *Pseudomonas* species to acquire and utilize exogenous fatty acids

Lipid exploitation is achieved by *P. aeruginosa* through key virulence factors delivered via Type III secretion. PopB and PopD mediate membrane lysis following interaction with the eukaryotic lipids cholesterol and phosphatidylserine [51, 52]. ExoU, a phospholipase A2 with affinity for phosphatidylinositol [53, 54], is one of several identified phospholipases that may be involved in chronic pulmonary infection and during pathogenesis of the cystic fibrosis lung [55–62]. In addition, phosphatidylcholine is prevalent in the mammalian lung and is targeted by *P. aeruginosa* during infection [63]. PC species in human lung surfactant contain linoleic and arachidonic acids, fatty acids not synthesized by *P. aeruginosa* [64], yet demonstrated to be incorporated into membrane phospholipids in this study. Other surfactant phospholipids, phosphatidylglycerol and phosphatidylinositol, also contain appreciable amounts of linoleic and arachidonic acid, and these fatty acid constituents are elevated in bronchoalveolar lavage samples from patients with respiratory distress [65]. The principle plasma phospholipid, PC 16:0/18:2, is recognized as a potential marker for uncompromised airways, since infiltration of this species correlates with severity of the asthmatic response [65]. It is tempting to speculate that host niche availability of phospholipids heightens accessibility to linoleic acid during *P. aeruginosa* infection of the CF lung. Surprisingly, the role of *P. aeruginosa* in the well-established fatty acid abnormalities in CF patients [66, 67] has not been investigated.

Measurement of permeability differed between crystal violet and ethidium bromide. Since crystal violet can bind to bacterial surfaces, the ethidium bromide assays constitute a better assessment of permeability caused by the PUFAs. We tested the accessibility of ethidium bromide by measuring both exclusion and accumulation of ethidium bromide. Both assays revealed similar trends, with dihomo-gamma-linolenic acid (20:3) eliciting the least membrane permeability. Interestingly, with the exception of 20:3, the accumulation assay correlated carbon number with permeability, with the 20-carbon PUFAs (arachidonic, eicosapentaenoic, and docosahexaenoic acids) causing elevated accumulation as compared to the 18-carbon PUFAs. Membrane permeability was not predictive of antimicrobial peptide resistance or biofilm formation, but it is intriguing to note that the observed decreases in motility correspond with permeability. Specifically, 20:3 exhibits little change whereas 20:4 and 20:5 exhibit significant changes compared to the control.

The effect of fatty acids on polymyxin B and colistin sensitivity are noteworthy. The results demonstrate that availability of several fatty acids raises the minimum inhibitory concentration of the cyclic peptide antibiotics tested, which are similar to cationic antimicrobial peptides (AMPs). These innate immune effectors were chosen because their activity is thought to rely upon intercalation in the membrane bilayer to eventually cause cell lysis [68]. Accordingly, assimilation of exogenous PUFAs into membrane phospholipids was hypothesized to alter AMP resistance. The most significant effects were observed with arachidonic acid, a prevalent fatty acid in eukaryotic phospholipids and mediator of cellular signaling and inflammation [69]. Rajamoorthi et al. [70] used deuterium NMR spectroscopy and small angle X-ray diffraction of n-6 (20:4) and n-3 (22:6) phospholipid bilayers to demonstrate distinct properties between the bilayers. Although both fatty acids confer membrane relaxation and reduced bilayer thickness, the n-6 (20:4)-containing bilayer exhibited a more disordered and deformable bilayer than the n-3 (22:6) bilayer [65]. Thus, contrary to our findings, it would be expected that incorporation of arachidonic acid might make the bacterial membranes more vulnerable to attack by AMPs. Another consideration for phospholipid alterations involves bacterial maintenance of membrane lipids with enzymes that change the fatty acids stereochemistry, such as cis/trans isomerase, a predicted periplasmic enzyme encoded by many *Pseudomonas* genomes [71]. Adoption of the *trans* conformation could explain both decreased permeability and heightened resistance to PMB and colistin observed in the current study. Furthermore, antibiotic attack relying on another mechanism (imipenem) did not significantly alter resistance. Utilization of another minimal medium (CM9) for polymyxin B yielded similar patterns although a 4-fold increase in resistance was observed with docosahexaenoic acid (22:6). Ongoing studies are defining the stereochemistry of phospholipids extracted from fatty acid exposed *P. aeruginosa*.

The link between pathogenicity and motility has been established for several pseudomonads, including *P. fluorescens* [72], *P. syringae* [73] and *P. aeruginosa* [74]. It is unknown whether PUFAs are chemotactic signaling molecules for *P. aeruginosa*, but the genomic possession of multiple gene clusters dedicated to chemotaxis suggests a wider repertoire of sensory capabilities [75]. Our findings of decreased motility in response to each PUFA may have ramifications for *P. aeruginosa* infection. Recently, swimming motility of *P. aeruginosa* was found to be instrumental in the formation of neutrophil extracellular traps, an important immune strategy to fight early infection [76]. A decrease in swimming motility in response to host fatty acids may contribute to early pathogenesis of *P. aeruginosa*. The fatty acid-mediated effects on swarming and twitching motility are under investigation. Importantly, we have not observed significant growth defects associated with fatty acids supplemented at a concentration of 300 μM (data not shown).

Biofilms contribute to the persistence of *P. aeruginosa* infection, and the impact of PUFAs on their formation has not been reported. The increase in biofilm production of the control culture from pH 5.5 to 7.4 correlates to previous studies of biofilm formation and pH in *P. aeruginosa* [77]; thus, our results suggest that exogenous PUFA availability may enable elevated biofilm formation in acidic conditions. *P. aeruginosa* biofilm cultures have been shown to contain a higher degree of long chain unsaturated fatty acids, but lower overall carbon length, as compared to planktonic cultures [78]. It is unknown whether, or to what extent, the exogenous fatty acids are assimilated in the biofilm cultures, as this was not analyzed in the current study. Further research is necessary to explore the connection between pH, biofilm formation and exogenous fatty acid utilization.

The hijacking of arachidonic acid from sites of infection could be advantageous for *P. aeruginosa* colonization and persistence. Assimilation of available PUFAs during infection may not only strengthen bacterial membranes, but also misguide the cellular responses reliant upon fatty acid signaling pathways. For example, the availability of unsaturated fatty acids increases macrophage phagocytosis of *P. aeruginosa* [79]. The anti-inflammatory and immunomodulatory effects of long chain polyunsaturated fatty acids have been documented [30, 32]. The administration of fatty acids during experimental and clinical *P. aeruginosa* infection has produced mixed results. While experimental studies have observed decreased mortality, decreased bacterial load and an anti-inflammatory immune response in mice [80], the human response is inconclusive. There is some precedent for the therapeutic potential of PUFAs. In two studies, the administration of n-6 PUFAs enhanced antibiotic activity against *P. aeruginosa* [81, 82]. Our results suggest that *P. aeruginosa* can achieve increased resistance to antimicrobial peptides when physiologically relevant exogenous fatty acids are available.

## Conclusions

Collectively, our findings emphasize the role that fatty acids may play in bacterial physiology, particularly for pathogens with the ability to utilize fatty acids for enhanced virulence and survival. The data herein demonstrate incorporation of several PUFAs into the phospholipids of *P. aeruginosa*. The exogenous PUFAs also affected membrane permeability, swimming motility and biofilm formation. Importantly, the presence of certain PUFAs increased the MICs of polymyxin B and colistin, suggesting a potential role of fatty acids in the pathogenesis of *P. aeruginosa* infections.

## Additional files

Additional file 1: **Figure S1.** Growth pattern of *Pseudomonas aeruginosa* in minimal media supplemented with individual fatty acids. Cultures of *Pseudomonas aeruginosa* were grown with or without 300 μM of the indicated fatty acids at 37 °C in G56 (pH 7.4) for 12 h. Cultures were inoculated at a starting OD_600_ of 0.05 and growth was assessed by measuring the absorbance (OD_600_) of the cultures every 40 min. (TIF 7504 kb)

Additional file 2: **Figure S2.** Growth pattern of *Pseudomonas aeruginosa* in the presence of exogenous fatty acids as the sole carbon source. Exogenous fatty acids (1 mM) were supplied in M9 minimal media (starting OD = 0.05; no glucose) as the sole carbon source and growth was monitored for 12 h at 37 °C. A positive control was included supplemented with 0.2% glucose. (TIF 9385 kb)

Additional file 3: **Figure S3.** Ultra performance liquid chromatography-mass spectrometry of isolated phospholipids from *Pseudomonas aeruginosa* grown in the presence and absence of fatty acids. Comparison of other fatty acids tested not shown in Fig. 2a (main text). All fatty acids tested showed altered chromatograms compared to the control. (TIF 3760 kb)

Additional file 4: **Figure S4.** Mass spectrometry of individual phospholipids from *Pseudomonas aeruginosa* grown in the presence and absence of fatty acids. Mass spectra for each of the peaks shown in Fig. 2b. The parent *m/z* is indicated and this is the value that was searched in the LIPID MAPS database. Identity of the specific phospholipid is indicated. Note that RT = retention time corresponding to the XIC in Fig. 2b. (TIF 21199 kb)

## Abbreviations

AMP: Antimicrobial peptide
CL: Cardiolin (diphosphatidylglycerol)
PE: Phosphatidylethanolamine
PG: Phosphatidylglycerol
PUFA: Polyunsaturated fatty acid
