# Supplementary figures and images for: Pseudomonas aeruginosa responds to exogenous polyunsaturated fatty acids (PUFAs) by modifying phospholipid composition, membrane permeability, and phenotypes associated with virulence
Source: BMC Microbiol. 2018 Sep 14;18:117. doi: 10.1186/s12866-018-1259-8 (PMC6137939; doi:10.1186/s12866-018-1259-8)

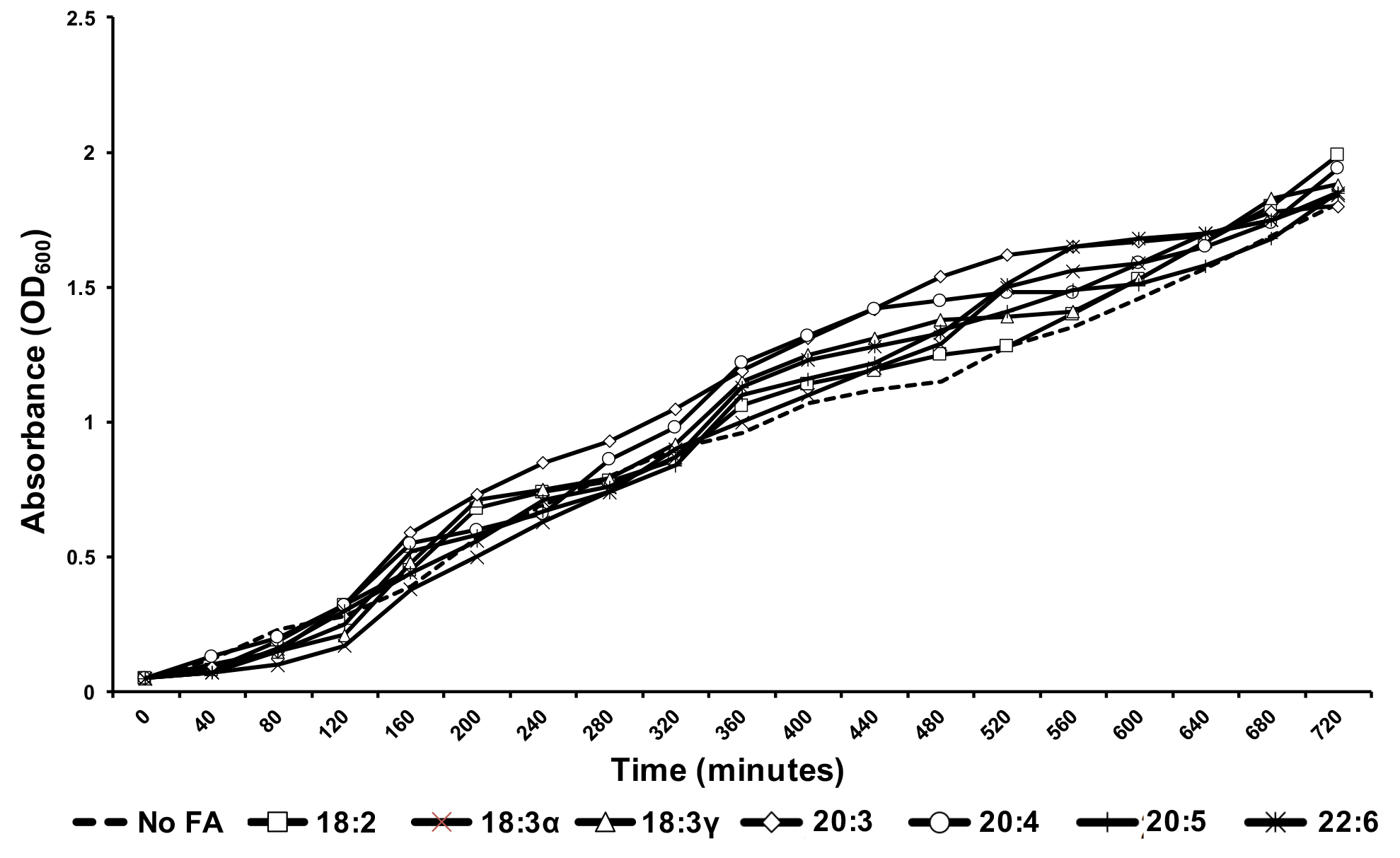

Supplement: Supplementary file 1 — Figure S1. Growth pattern of Pseudomonas aeruginosa in minimal media supplemented with individual fatty acids. Cultures of Pseudomonas aeruginosa were grown with or without 300 μM of the indicated fatty acids at 37 °C in G56 (pH 7.4) for 12 h. Cultures were inoculated at a starting OD600 of 0.05 and growth was assessed by measuring the absorbance (OD600) of the cultures every 40 min. (TIF 7504 kb) [file 12866_2018_1259_MOESM1_ESM.tif]

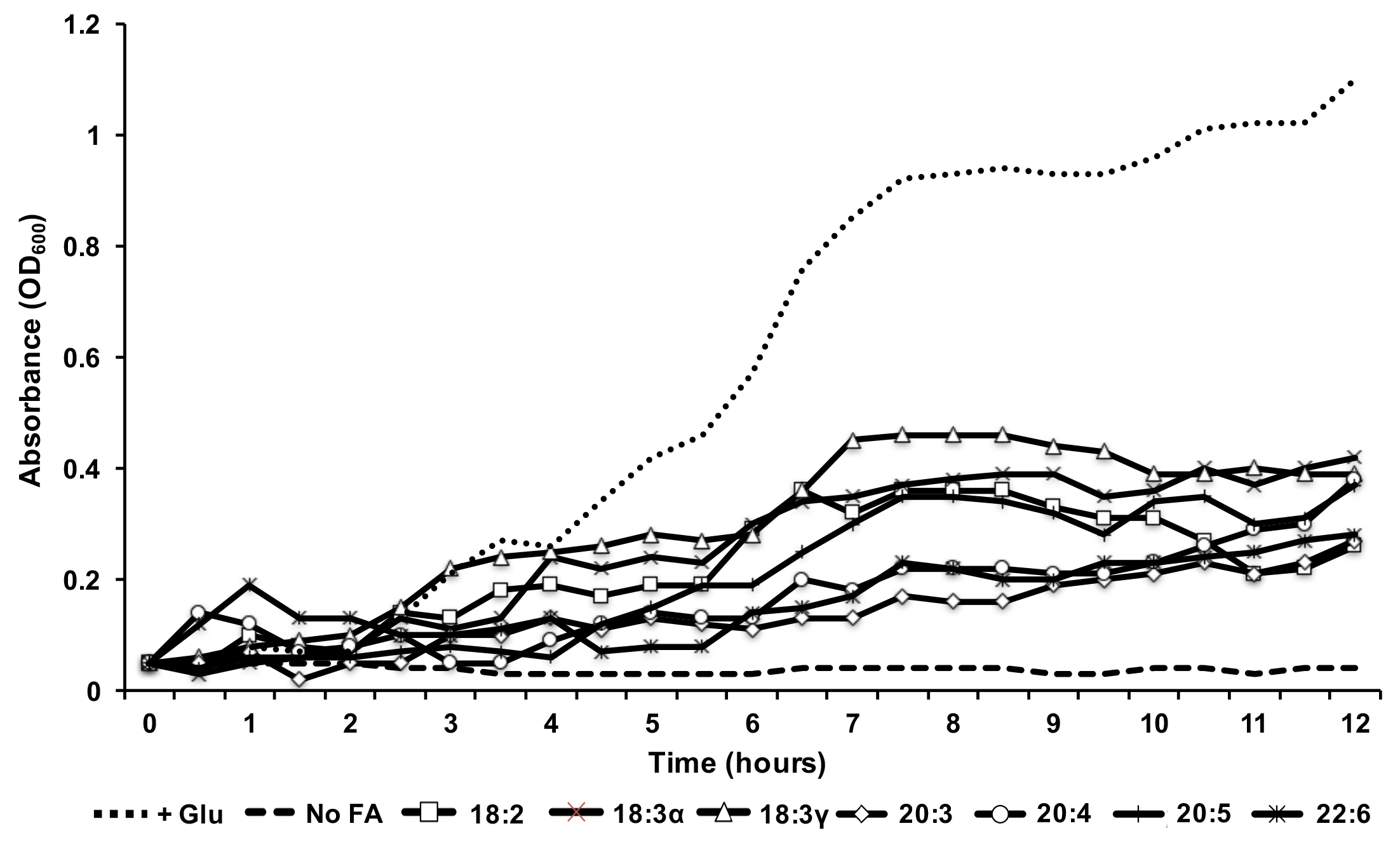

Supplement: Supplementary file 2 — Figure S2. Growth pattern of Pseudomonas aeruginosa in the presence of exogenous fatty acids as the sole carbon source. Exogenous fatty acids (1 mM) were supplied in M9 minimal media (starting OD = 0.05; no glucose) as the sole carbon source and growth was monitored for 12 h at 37 °C. A positive control was included supplemented with 0.2% glucose. (TIF 9385 kb) [file 12866_2018_1259_MOESM2_ESM.tif]

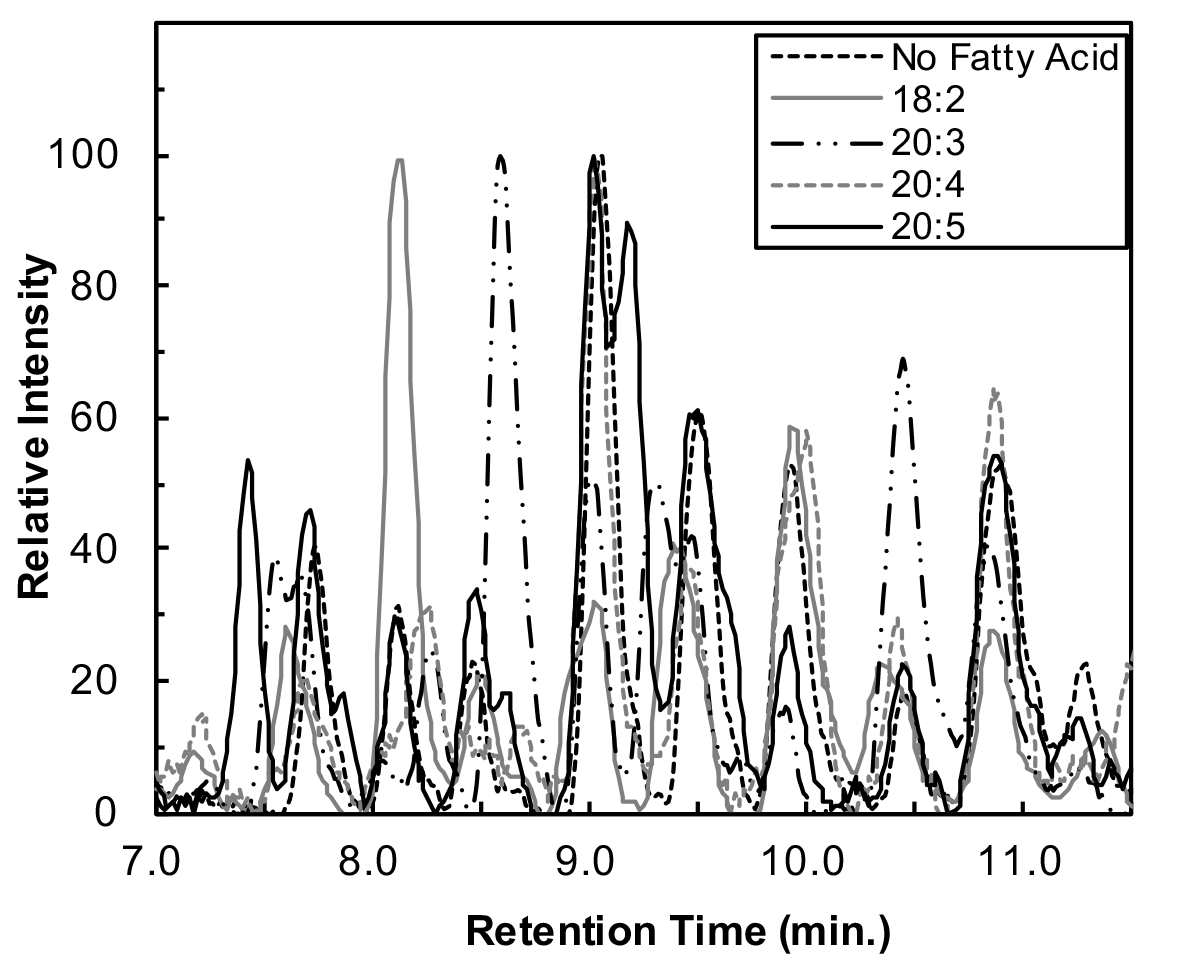

Supplement: Supplementary file 3 — Figure S3. Ultra performance liquid chromatography-mass spectrometry of isolated phospholipids from Pseudomonas aeruginosa grown in the presence and absence of fatty acids. Comparison of other fatty acids tested not shown in Fig. 2a (main text). All fatty acids tested showed altered chromatograms compared to the control. (TIF 3760 kb) [file 12866_2018_1259_MOESM3_ESM.tif]

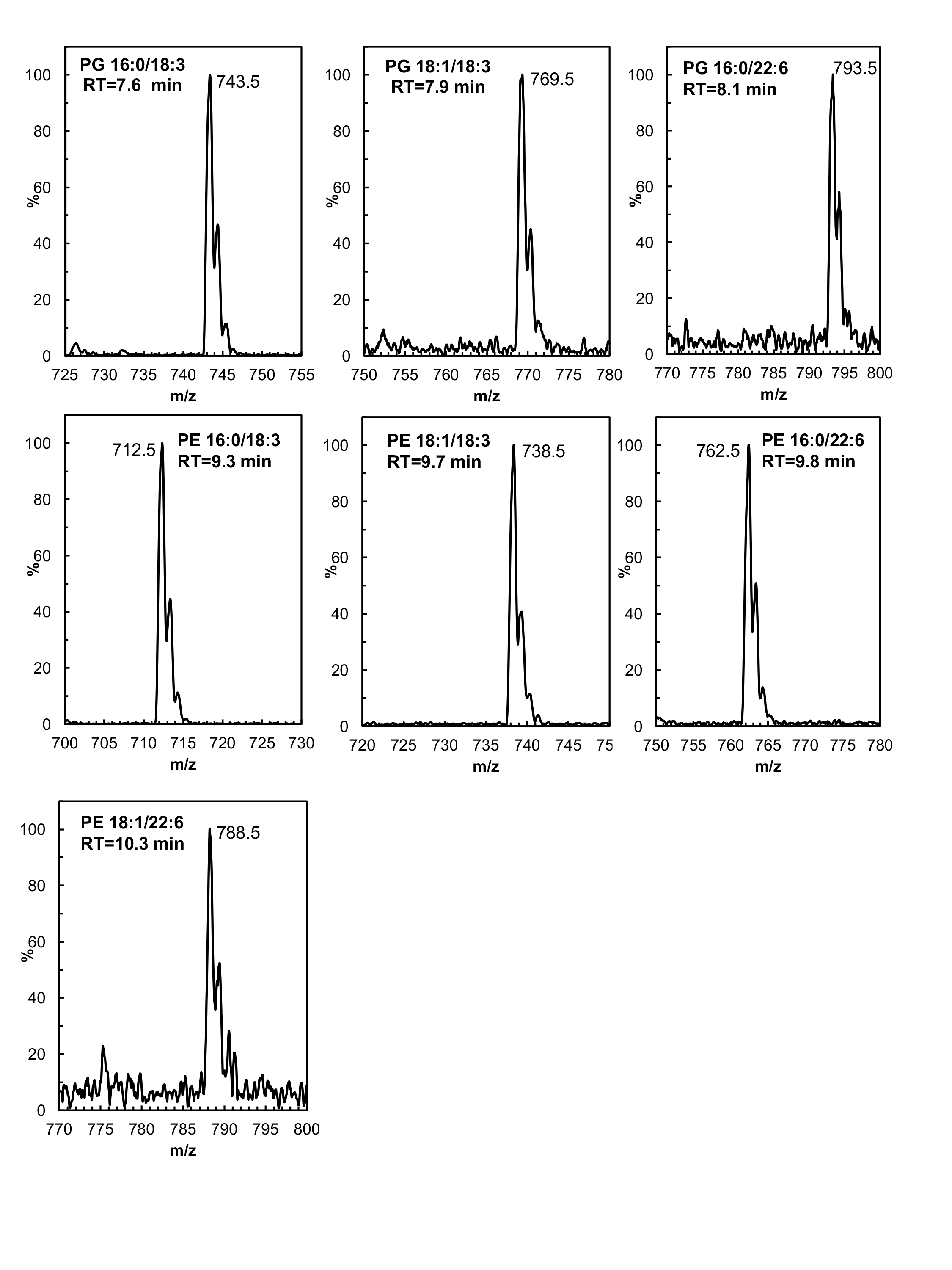

Supplement: Supplementary file 4 — Figure S4. Mass spectrometry of individual phospholipids from Pseudomonas aeruginosa grown in the presence and absence of fatty acids. Mass spectra for each of the peaks shown in Fig. 2b. The parent m/z is indicated and this is the value that was searched in the LIPID MAPS database. Identity of the specific phospholipid is indicated. Note that RT = retention time corresponding to the XIC in Fig. 2b. (TIF 21199 kb) [file 12866_2018_1259_MOESM4_ESM.tif]
